# Industrial Energy Assessment Training Effectiveness Evaluation: An Eye-Tracking Study

**DOI:** 10.3390/s21051584

**Published:** 2021-02-24

**Authors:** Laleh Ghanbari, Chao Wang, Hyun Woo Jeon

**Affiliations:** 1 Bert S. Turner Department of Construction Management, Louisiana State University, 3319 Patrick F. Taylor Hall, Baton Rouge, LA 70803, USA; lghanb1@lsu.edu; 2 LSU-Industrial Assessment Center, Louisiana State University, 3131 Patrick F. Taylor Hall, Baton Rouge, LA 70803, USA; hwjeon@lsu.edu; 3 Department of Mechanical & Industrial Engineering, Louisiana State University, 3261 Patrick F. Taylor Hall, Baton Rouge, LA 70803, USA

**Keywords:** training effectiveness, industrial energy assessment, eye-tracking, energy efficiency, visual attention behavior

## Abstract

It is essential to understand the effectiveness of any training program so it can be improved accordingly. Various studies have applied standard metrics for the evaluation of visual behavior to recognize the areas of interest that attract individuals’ attention as there is a high correlation between attentional behavior and where one is focusing on. However, through reviewing the literature, we believe that studies that applied eye-tracking technologies for training purposes are still limited, especially in the industrial energy assessment training field. In this paper, the effectiveness of industrial energy assessment training was quantitatively evaluated by measuring the attentional allocation of trainees using eye-tracking technology. Moreover, this study identifies the areas that require more focus based on evaluating the performance of subjects after receiving the training. Additionally, this research was conducted in a controlled environment to remove the distractions that may be caused by environmental factors to only concentrate on variables that influence the learning behavior of subjects. The experiment results showed that after receiving the training, the subjects’ performance in energy assessment was significantly improved in two areas: production, and recycling and waste management, and the designed training program enhanced the knowledge of participants in identifying energy-saving opportunities to the knowledge level of experienced participants.

## 1. Introduction

Organizations invest mostly in enhancing their employees’ knowledge and skills; based on the training industry magazine report, the training expenses on employees have increased by 16% from 2018 to 2019 [1]. Therefore, organizations need to understand the effectiveness of their training program and determine the rate of investment return. Besides, it would be a good indicator of future education to identify the knowledge level of the trained employees. By measuring the knowledge level of trainees after being trained, it can find out which training sections should be improved in the future. Companies need to have a reliable procedure to identify any progress made on employee’s performance after the training. One way of discovering the changes in trainees’ knowledge is to evaluate their visual behavior, which reflects on their attentional allocation; therefore, eye-tracking study becomes to be a popular means of measuring attention because there is a high correlation between where one is looking at and where they are focusing their attention on [2,3].

Neuro-physiological devices such as eye-trackers can provide more accurate data related to human behavior than self-reported methods such as survey questionnaires because the eye-trackers can capture the eye-movement of participants in experiments and reflect their evaluations. Eye-tracking devices and technology have been widely adopted to effectively capture and quantify users’ selective visual attentional behaviors [4], however, it has been limited studies on measuring training effectiveness, especially for industrial energy assessment training.

According to the U.S. Energy Information Administration (EIA), about 51% of the energy consumed in the industrial sector is wasted [5]. The Industrial Assessment Center (IAC) is a USDOE-funded technical assistance program that has specifically focused on helping small and medium-sized manufacturing facilities cut back on unnecessary costs from inefficient energy use since 1978 and was later expanded to include evaluations of ineffective production procedures, excess waste production, and other production-related problems. The industrial assessments are performed in the facility within one day by the IAC center engineering faculties with the upper-class undergraduate students and graduate students. To be qualified for an industrial assessment on the field, all faculties and students have to go through the proper training in the class. The objective of this study is to investigate the effectiveness of the designed industrial energy assessment training program and to identify the relationships among energy assessment knowledge, attention, and ability in detecting the potential energy-saving opportunities through monitoring the visual attentional behavior using eye-tracking technology.

### 1.1. Training Program Evaluation

To evaluate the effectiveness of a training program, the defined indicators play an essential role. Previous studies introduced different measures for evaluating the training program. Ghosh et al. [6] discussed the characteristics of trainers that influence the effectiveness of a lecture-based training program. They collected data by administering a questionnaire to 80 employees. Data analysis indicated that the knowledge level of the trainer and trainer’s interpersonal skills were the most critical factors that affect the effectiveness of training programs. Another study by Ghosh et al. [7] evaluated the satisfaction of trainees as the indicator for determining the effectiveness of the training program. They measured the satisfaction level based on six factors: the venue of the program, food served, communication of the trainer, practical application, other facilities, and clarity of the trainer. Ostroff [8] demonstrated that measures that consider typical situations that workers may confront on the job would better indicator for evaluating training program effectiveness. Thus, cultural impact, efficiency impact, and financial impact were recognized as measures for assessing the effectiveness of a training program. Efficiency impact is a metric that can be defined in various ways based on the industry that is using a training program. For instance, in assessing the effectiveness of a training program for the energy industry, the efficiency can be measured based on the trainees’ attention to the energy-saving opportunity before and after receiving the training program.

In addition to identifying the best indicator for evaluating the effectiveness of the training program, recognizing the right tool for evaluating training is significantly essential. Each tool can be used for a specific purpose. To evaluate the effectiveness of the training program based on the emotional reaction of trainees, post-course questionnaires are standard. Pre- and post-tests are useful tools to measure learning objectives as the indicators for evaluating the effectiveness of the training program. Surveys, interviews, and observations can be applied to assess behavioral changes as the measures of determining the effectiveness of the training program. Finally, the impact on the organization as an indicator for assessing the effectiveness of a training program can be measured by recording the performance before and after receiving the training [9]. Performance can be recorded using different methods. One of the novel technologies that can be used to measure performance is eye-tracking technology, which monitors visual behavior.

### 1.2. Application of Eye-Tracking Technology

The robust relationship between cognitive behavior and eye movement pattern is the reason for using eye-tracking technology in various fields. In the field of psychology, researchers have used psychological tests to discover individuals’ intelligence and social attributes. Pettersson et al. [10] discussed the cognitive behavior of individuals in the Raven Progressive Matrices (RPM) test. They applied virtual reality technology to simulate the experiment environment and monitored the eye-movements of experiment participants using an eye-tracker to analyze and measure their ability in reasoning. Mark et al. [11] did a combined psychological–neurological experiment on twenty-three subjects to assess their improvement during four weeks of training in a six-task cognitive battery. They measured the participants’ eye movement in addition to other psychological criteria such as heart rate as indicators of the subjects’ performance to prove that, by increasing in skill level, the workload related to vigilance skill could be increased over time while the starting workload was decreased. In the healthcare field, Davies et al. [12] conducted a study on the thirty-one medical staff who were responsible for the interpretation of electrocardiograms (ECGs) to find the effect of age and experience on individuals’ ability to interpret an ECG scan. They used an eye-tracker to record the gaze behavior of participants and applied it as a measure of interpretation accuracy. Many studies have also been conducted in the field of safety; Desmet and Diependaele [13] examined the effect of hands-free phoning on the visual attention of drivers by analyzing the eye movement patterns of drivers recorded by the eye-tracker while hands-free phone calling. It showed that drivers’ fixation on the road signs, other vehicles, and the speedometer was reduced when they talked on the phone. Additionally, drivers have a broader spatial distribution of eye fixation during a call. Kircher et al. [14] performed another study to evaluate the performance of truck drivers after receiving training, and the recorded visual behavior by a head-mounted eye-tracker showed that the truck drivers paid more attention to cyclists after receiving the training and had much better speed management. Besides driving safety, workers’ attentional behavior toward safety problems on construction sites was also highly related to their personality, according to the study through tracking workers’ visual behavior by Hasanzadeh et al. [15], it showed that those who were introverted, conscientious, and open to experience could respond to hazardous areas more efficiently. Another study by Hasanzadeh et al. [16] showed that work experience and injury exposure could significantly increase the construction workers’ hazard detection capability, and they also stated that Occupational Safety and Health Administration (OSHA) 10-hour certificate training was not effective in enhancing workers’ attention to hazard detection through monitoring the visual behavior of participants using an eye-tracker. Understanding consumers’ visual attention could help designers update their design or product to better fit the consumers’ demands. Dupont et al. [17] discussed how the urbanization level of a landscape could influence the ability of individuals to explore it. Their study results showed that there was a direct correlation between complex urbanized landscapes and the time required to explore them visually. Ahn et al. [4] discussed the eye movement of ninety-seven online shoppers, using an eye-tracker to capture their areas of interest during e-commerce. The results showed an exponential reduction in customers’ attention when they went through from the top to bottom of a search result webpage—additionally, the total number of purchasing options influences this decay significantly. Mohammadpour et al. [18] conducted a study to investigate the reaction of building end-users to a façade designed by the architects. They used an eye-tracker to evaluate end-users’ visual behavior when they were watching four 3D-models. Their study showed that the design alternatives with a high level of users’ satisfaction attracted attention considerably. Wang et al. [19] examined students’ behavior in learning cooking recipes, and they requested twenty-nine volunteers to read static (text and picture) and dynamic (text and video) recipes and monitored their eye movement with an eye-tracker. The results showed that students paid more attention to the text compared with a picture on a static page. Netzel et al. [20] conducted a map reading test with forty participants to find out the best design for map layouts. By analyzing their visual scanning strategies, the results showed that individuals could read the color maps more correctly and faster than the gray-scale maps. Fotios et al. [21] explored the optimum level of lighting for sidewalks through analyzing the gaze behavior, and their results indicated that lighting intensity should be enough for: (i) visibility of distant people to find their intent to be informed about route direction; and (ii) visibility of near path to inspect the surface for finding the best place for foot (less than 4 m). In the field of computer vision, researchers have also used eye-tracking technology to help with object detection from a video clip. Shanmuga Vadivel et al. [22] presented an algorithm to automatically extract salient objects in videos through monitoring the visual behavior together with machine learning technologies.

Table 1 shows some of the metrics that have been used in previous studies for evaluating visual behavior. The gaze location shows the position where the eyes are looking, and pupil dilation is the actual, internal and, physical size of the pupil which is correlated with the level of cognitive load that the user is experiencing. The most common parameter for evaluating visual behavior is the fixation duration that records the time when the eye ceases scanning about a scene so that the visual system can collect data about what is being observed. Another metric is fixation frequency, which is the number of times that the user returns their attention to an area of interest. The other standard measurement for tracking eye behavior is saccade. A saccade is a quick eye movement from one location to another [23]. It can be measured based on the duration, which is called the saccade duration, or based on the number of times, which is called saccade frequency. Total recording duration is another parameter that has been frequently used for measuring attentional behavior, and it stands for the time recorded while the eyes are focusing on the same location. The last metric identified from the literature related to monitoring visual behavior is the first fixation duration, which means how long the first fixation lasts.

### 1.3. Point of Departure

There is a high correlation between attentional behavior and where one is focusing on; therefore, various studies have applied standard metrics for the evaluation of visual behavior to recognize the areas of interest that attract individuals’ attention. Training can enhance the knowledge of a person related to areas of interest, but attention is necessary to recognize and understand them appropriately. In this study, students required training to be qualified for an industrial energy assessment, and by evaluating the attentional behavior of students after receiving the training, the effectiveness of the training program was evaluated so it could be improved accordingly.

However, through reviewing the literature, we believe that studies that applied eye-tracking technologies for training purposes are still limited [14], especially in the industrial energy assessment training field. This proposed study departs from the current body of knowledge, because it is one of the earliest studies that investigate the effectiveness of an industrial energy assessment training program using eye-tracking technology. Moreover, this study identifies the areas that require more focus based on evaluating the performance of subjects after receiving the training. Additionally, this research was conducted in a controlled environment to remove the distractions that may be caused by environmental factors to only concentrate on variables that influence the learning behavior of subjects. Therefore, the objective of this paper is to evaluate the performance of students in recognizing energy-saving opportunities after receiving in-class training, and the evaluation results is further analyzed to assess the effectiveness of the industrial energy assessment training provided.

## 2. Materials and Methods

In this study, a novel evaluation procedure for investigating the effectiveness of training methods about the performance of students in facility energy assessment was developed. Training content was collected from a combination of field energy assessment experience, management concerns, IAC training materials, and general industry knowledge from industrial energy assessment experts. The designed experiment was divided into four sections: (1) collecting data on subjects about their attentional allocation behavior to potential energy-saving opportunities before the training; (2) training subjects in the class environment; (3) testing the trainees’ knowledge level after the training using a traditional quiz; and (4) evaluating the subjects’ performance on identifying the energy-saving opportunities after the training session. Before starting the experiment, all the experimental procedures were approved by the Institutional Review Board (IRB) at Louisiana State University (IRB# 4293).

### 2.1. Participants

Sixteen college students (two females, fourteen males) from Louisiana State University participated in the experiment. They were at different levels of the school year (four graduates, five juniors, four seniors, and three sophomores). All the participants were young, with a minimum age of 20 years old and a maximum age of 31 years old (25% equal or more than 25 years old, 75% less than 25 years old). Of the participants, 31% had (one year or more) experience in energy assessment activities, and 69% of them did not have any work experience in energy assessment and had not received any training in energy assessment (in-class or in-field). Before starting the experiment, participants were required to fill out a questionnaire regarding their medical history (e.g., eye problems) as well as experience and training in energy assessment activities. For analyzing data, at first, data collected through questionnaires were evaluated and those data related to participants who suffered from eye problems were removed. In the next step, to categorize participants, two factors were used: experience, and training. Thus, students who had not received any training (in-class or on-field) and did not have any energy assessment experience were in one group, A1, students who received the in-class training were in group A2 and, experienced participants were in the group A3.

Based on the participants’ medical history data, one of the participants did not meet the requirement for experimenting. Therefore, data related to that student were removed, and the analysis of data was performed based on data of fifteen participants who did not have any medical issues (ex. eye movement abnormalities, eye surgery). Eye problems may cause issues in the accuracy of the collected data. Finally, 75% of subjects who did not have any work experience in energy assessment or receive any prior training in this area received in-class training in the experiment.

### 2.2. Eye-Tracking Device

**Figure 1:**
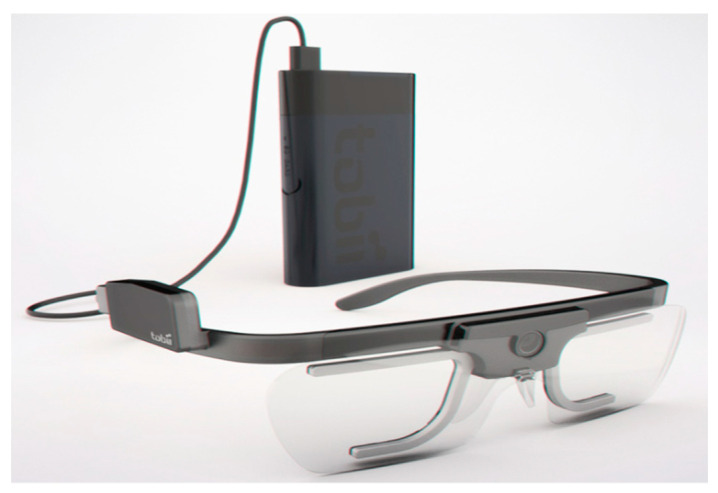
Tobii Pro Glasses 2 used as the eye tracker in this study [27].

Tobii Pro Glasses 2 (manufactured by Tobii AB Company, Danderyd Municipality, Sweden) were used in this study to capture the visual behavior of participants. This eye-tracker has a high spatial resolution and a sampling rate of 100 Hz and contains four cameras (two for each eye) that can record the movement of each eye. The eye-tracker glasses were calibrated on each subject during each section of the experiment. As shown in Figure 1, the eye-tracker has an excellent ability to support automatic slippage compensation. This means that slippage of head units, which is unavoidable during a study, does not have an impact on the quality of data, because the eye-tracker remains calibrated using a gyroscope and accelerometer sensors.

### 2.3. Scenario Images and Areas of Interest

**Figure 2:**
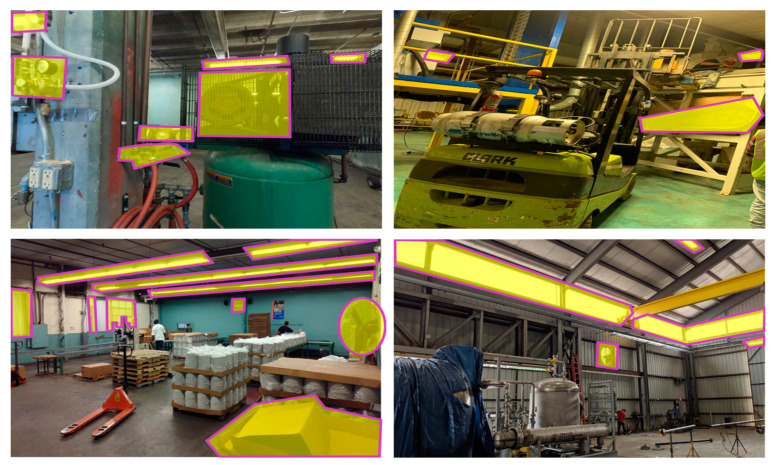
AOIs are highlighted for the experiment.

To prepare images for the experiment, we collected around 2000 images of energy-saving opportunities from several real facilities during the field industrial energy assessments. Then, certified practitioners screened these images and selected 40 images with 96 areas of interest (AOIs) as potential energy-saving opportunities. AOIs could be located in one of the following categories: production (furnace, oven, boiler, pump, chiller, cooling tower, motor system, air compressor), building and HVAC (building envelope insulation, lighting, HVAC system, skylight, occupancy sensor, thermostat), and recycling and waste management (recycling baler, recyclable material, anaerobic digestion of wastewater sludge). As shown in Figure 2, AOIs within each image have been manually predefined by the industrial energy assessment experts to capture the attention of each AOI, in Figure 2, each highlighted area is an individual AOI for data collection.

The size of AOIs has a significant impact on the accuracy of the analysis. Holmqvist et al. [28] analyzed the size of AOIs, and they advised to keep the maximum size for AOIs to include all fixations which belong to an object. Orquin et al. [29] discussed the impact of AOI size on the measurement of object attention and conclusion about cognitive processes. They reanalyzed four published studies to find the optimal size of AOIs. They suggested that, in studies with overlapped fixation distribution (smaller distance between objects), smaller AOI sizes would produce the best results. In comparison, when there is a more considerable distance between objects, it is recommended to use larger AOIs. They explored an AOI margin of 0.5° of visual angle for the best-fitted model of overlapped fixations. At the same time, the primary studies on AOI sizes prescribed a visual angle margin of 1–1.5° for all experiments [28]. It was suggested in experiments with the freedom to design stimuli, that the maximum distance between objects would reduce the overlapping of fixation distribution as well as error in the analysis [29].

### 2.4. Data Collection

**Figure 3:**
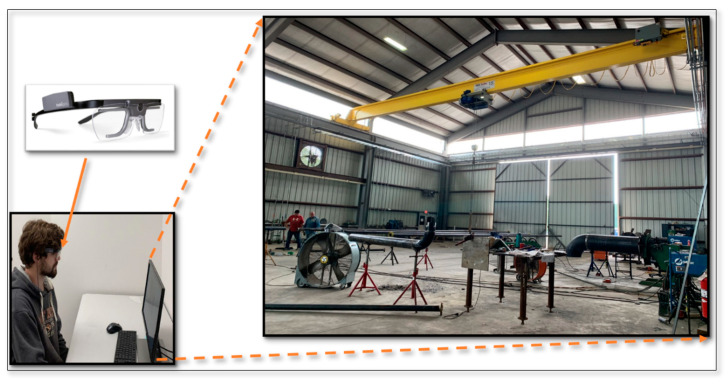
Lab setting for the experiment.

Before the experiment started, students were asked to answer questions about their experience, certificate, and training in energy assessment activities. Moreover, because this study was designed based on eye-movement tracking, it should be confirmed that participants did not have eye problems. Therefore, participants were required to answer some questions related to their medical history before starting the test. In the first step of the experiment, students had to look at each image shown on the monitor of a desktop computer for 15 s and look for potential energy-saving opportunities (Figure 3). Participants were seated approximately 45 cm from the monitor. Simultaneously, a wearable eye-tracker was used to capture participants’ visual attentional behavior. After each image, students had to answer a question about the amount of energy-saving opportunities they identified in the image. In the second section of the experiment, students who did not have any training, experience, or certificate in energy assessment (group A1) attended a training program that was hosted by a certified energy efficiency practitioner. Training topics were categorized into three sections: recycling and waste management, production, and buildings and HVAC systems. The first training part included energy-saving opportunities related to wastewater and recyclable material. The content of the second part was about process heating systems such as boilers, process cooling systems such as chillers, and motor systems such as air compressors. In the last section of training, material related to energy-saving opportunities for lighting, HVAC systems, and building insulation was presented. During the training program, students were taught to look for what type of information related to each energy-saving opportunity. They were trained about the essential information needed to be collected during an energy assessment session. Multiple images from the real facility were shown to students during the training program to familiarize them with energy-saving opportunities in the facilities.

In the third step, the enhancement of participants’ knowledge about energy assessment topics was investigated. Students were required to take a quiz about training content. The goal of this quiz was to understand whether students had learned what has been taught during the training. The results of this phase helped us to prune participants for the final stage of the experiment. It means that participants who could not achieve the acceptable grade (80%) in this test were not qualified for the final step of the experiment. In the last phase of the study, the energy assessment performance of participants after being trained was evaluated. Therefore, trainees were requested to identify potential energy-saving opportunities from images shown on the monitor while the eye-tracker recorded their visual attentional behavior. Participants looked at each picture for 15 s; then, they answered a question about the amount of penitential energy-saving opportunities they identified in the image. It should be noted that the same images were used for the first and last sections of the experiment.

### 2.5. Data Analysis

In this study, data collection was performed based on the requirements to test two hypotheses (Table 2). Two types of variables were considered: performance in energy assessment (energy assessment knowledge, i.e., training, experience, and a certificate in energy assessment) as the independent variable and eye-tracking metric (fixation) as the dependent variable. Data related to the independent variable were gathered through a questionnaire before starting the experiment. Participants responded to questions such as whether they had work experience related to energy assessment, or whether they had any energy assessment certificate, or whether they had received any energy assessment training (in-class or in-field). Data related to the dependent variable were collected using the eye tracker. In this study, one of the common fixation-related metrics-the fixation duration, was analyzed. Data provided by the eye tracker were screened based on the students’ responses to questions related to the amount of energy-saving opportunities identified from the image. These questions were displayed on the screen after showing each image. For example, if there were two potential energy-saving opportunities in the image but the student only found one, data related to one opportunity were used for analysis.

In the experiment, participants were separated into groups based on the collected data from the questionnaire to test the hypotheses. For hypothesis 1, the performance of group A1 and group A2 were compared and, for hypothesis 2, group A2 and group A3 were compared.

In this study, the performance of energy assessment was evaluated based on the participant’s visual attentional behavior during the experiment. We conducted the data analysis in the data analytics tool (Tobii Pro Lab) provided with the eye tracker, which automatically mapped the collected eye movement data to the corresponding AOIs. Then, within each AOI, the eye-movement data were further analyzed to obtain the fixation duration data of each participant based on the data provided by the eye tracker and participants’ responses to questions displayed after each image. The eye tracker recorded the locational patterns of eye movement, which were finally analyzed to generate a graphical representation of the visual behavior of participants, such as gaze plots and heat maps.

The main goal of this study was to evaluate the effectiveness of the designed training program in enhancing students’ performance during an energy assessment. Therefore, the performance of participants before and after the training session was compared. A Student’s *t*-test was adopted to compare the average of two groups. Several requirements, such as independent subjects, normality, and homogeneity of variances, must be satisfied for using a *t*-test [30]. Independency of subjects means that no item or subject can belong to two groups simultaneously. Normality means measured data in the experiment should be approximately normally distributed. In this study, a Shapiro–Wilk’s W test was applied to test the normality of datasets, because the number of samples was small. Homogeneity of variances means that variances are equal across the groups which are compared. Regarding the homogeneity of variances, Levene’s test, which considers the null hypothesis that, “the variance is equal across the groups which are compared” [31] was used. Homogeneity of variances can be ignored if the number of subjects for two groups that are compared is the same.

## 3. Results and Discussions

The performance of participants in the energy assessment was divided into three categories (recycling and waste management, production, buildings and HVAC) based on the in-class training material. Data related to the first hypothesis are shown in Table 3, which tests the impact of in-class training on the performance of students in the energy assessment3. Table 4 shows the result of the experiment for hypothesis two, which compared the visual attentional behavior of two groups of participants (experienced students (A3) vs. students who received in-class training (A2)) in recognizing energy-saving opportunities.

In this study, the participants were separated into three groups for comparison: pre-training group, post-training group, and experienced group. These groups have completely independent subjects. The first group were participants who did not have any experience or receive any training (in-class or on-field) related to industrial energy assessment; the subjects of group A1 were independent of the second group subjects who received the designed in-class training. On the other hand, subjects of the third group were experts with in-field experience. Therefore, the subjects of the experiment were different and unrelated and satisfied the requirement (independent subjects) for the *t*-test. Next, we further analyzed the data from the perspectives of independent subjects, normality, and homogeneity of variances to ensure the data collected meet the requirement of the Student’s *t*-test.

Regarding the normality, because the number of samples was not large, the Shapiro–Wilk’s W test (at the significance level of 0.05) was used to test the normal distribution of each group separately. Table 5 shows the results of Shapiro–Wilk’s W test. Based on the provided result, *p* > 0.05 for each group individually, the null hypothesis (the data is normally distributed) was retained, and normality could be assumed for the dataset.

Table 6 indicates the results of Levene’s test (at the significance level of 0.05). It should be noted that because of the equal sample sizes, Levene’s test was not performed between group A1 and group A2. Based on the results provided by Levene’s test, the *p*-value is more than 0.05 in all experiments. Therefore, the null hypothesis was retained, and equality of variances across the groups was assumed.

As analysis is shown above, three requirements (normality, independent subjects, and homogeneity of variances) for using a parametric test were satisfied. Therefore, we applied the *t*-test (at a significant level of 0.05) to compare the energy assessment performance of subjects who participated in the experiment (Table 7). In the first analysis, the visual attentional behavior of subjects, before and after training, was discussed based on three categories: production, building and HVAC, and recycling and waste management. A total of 75% of participants in the experiment received in-class training. These subjects did not have any work experience in energy assessment. They also had not received any training (in-class or in-field) related to energy assessment activities before the current experiment. Results showed that the attentional behavior of students to energy-saving areas related to production increased after the training program (*t* = 2.465. *p* = 0.011 < 0.05). Therefore, the training program had a positive effect on enhancing the subject’s knowledge about energy assessment related to production areas. Regarding building and HVAC areas, performance of students did not change significantly after training (*t* = 0.817, *p* = 0.211 > 0.05). Therefore, the null hypothesis could not be rejected in this test, and it was assumed that the participant’s attentional behavior to energy-saving opportunities related to building and HVAC was similar before and after training. It can be concluded that training contents need to be improved in the area of building and HVAC. Regarding the recycling and waste management area, *t*-test results indicated that the knowledge of subjects after training increased (*t* = 1.721, *p* = 0.05). Therefore, contrary to the null hypothesis, the training program had a significant positive impact on trainees’ ability to recognize energy-saving opportunities related to the area of recycling and waste management.

The second analysis compared the performance of students who received the in-class training (group A2) with the performance of experienced subjects (group A3) in energy assessment. Of these, 25% of participants in this study had one year or more experience in energy assessment activities, and 75% of subjects received the in-class training; those who had not any experience related to energy assessment and had not received any training (in-class or on-field) in this area. The results showed that for the production area, the null hypothesis could not be rejected (*t* = 0.585, *p* = 0.568 > 0.05) and equality of performance of students who received the training program (group A2) with the performance of experienced participants (group A3) in energy assessment was retained at a significance level of 0.05. Therefore, it showed that the training program was good enough to enhance the knowledge of participants to the level of experienced subjects’ knowledge in recognizing energy-saving opportunities in the production area. Regarding building and HVAC area, Group A2 and group A3 demonstrated the same performance (*t* = 0.127, *p* = 0.9 > 0.05). Therefore, the null hypothesis was retained at a significance level of 0.05 in this test. Considering the result of the first hypothesis, it could be concluded that neither training nor experience has a significant impact on participants’ visual attentional behavior in recognizing energy-saving opportunities in the area of building and HVAC. Regarding recycling and waste management, the results showed that the performance of students who received the training (group A2) was not the same as the performance of experienced subjects (group A3) (*t* = 2.275, *p* = 0.04). It means that the null hypothesis was rejected (at a significant level of 0.05) in favor of the alternative hypothesis (performance of the students who received the training (group A2) was not equal to the performance of students who were experienced (group A3)). Therefore, the research team decided to consider another alternative hypothesis to check whether the students who received the in-class training (group A2) showed better or worse performance compared with experienced subjects (group A3). Results indicated that, after the training, students demonstrated better performance in recognizing energy-saving opportunities in the area of recycling and waste management compared with experienced subjects (*t* = 2.25, *p* = 0.02). Therefore, the training program was effective in this area.

This study intended to use the results to identify whether there was a noticeable and measurable positive change in the performance of participants in energy assessment after receiving training. Therefore, the visual attentional behavior of participants was monitored during the experiment to investigate their progress in recognizing potential energy-saving opportunities. The first null hypothesis concerned comparing participants’ performance in energy assessment before and after receiving the in-class training. The results showed that participants’ knowledge in two areas (production, recycling, and waste management) increased significantly after receiving the training.

Regarding the building and HVAC area, a significant difference in the visual attentional behavior of participants was not observed. Considering the content of the training program in the building and HVAC area, it could be concluded that participants were familiar with the presented topics even before receiving the training program because, in this area, the covered material for training was about lighting, HVAC, doors, and windows. The results demonstrated that the training program was successful in enhancing subjects’ knowledge about recognizing energy-saving opportunities.

The second null hypothesis was about comparing the energy assessment performance of participants who received the in-class training with the performance of participants’ who were experts with in-field assessment experience. Experimental results indicated that participants who received the in-class training had the same performance or even better than experienced people. Regarding production areas, training content was successful in enhancing the participants’ knowledge to the knowledge level of experienced people. It means that, after the training, participants who received the in-class training showed the same performance in energy assessment comparing with experienced subjects. Regarding the area of building and HVAC, the performance of experienced participants and participants who received the in-class training was the same in recognizing energy-saving opportunities. In the field of waste management and recycling, the application of training content was fantastic; the result showed that subjects who received the in-class training had better performance in recognizing energy-saving opportunities compared with people who were experienced. Therefore, based on data analysis, the energy assessment training program was effective in enhancing students’ knowledge.

By comparing the post-training performance of students with the performance of experienced and certified energy practitioners, it can be determined whether a trainee is qualified to become a certified energy practitioner. All the experienced people who participated in this study were certified energy practitioners, therefore comparing the performance of experienced participants (group A3) with subjects who received the in-class training (group A2) was similar to comparing the performance of energy certified practitioners and participants who received the in-class training (group A2). Based on the data analysis, subjects who received the in-class training had enough knowledge to become energy-certified practitioners because they demonstrated the same performance or even better in recognizing energy-saving opportunities compared with people who possessed energy certifications.

The experienced participants of the current study had not received any in-class training before performing the experiment, and they gained experience during attending energy assessment sessions on the field. Therefore, comparing the performance of students who received the in-class training (group A2) with the performance of experienced participants (group A3) also provided useful information about enhancing knowledge in energy assessment through two different methods (in-class and in-field). The students who received the in-class training (group A2) demonstrated the same or better performance in recognizing energy-saving opportunities compared with participants who obtained energy assessment knowledge through attending the real facilities. Therefore, it can be concluded that the in-class training was as positive as the training in the real environment of a facility.

**Figure 4:**
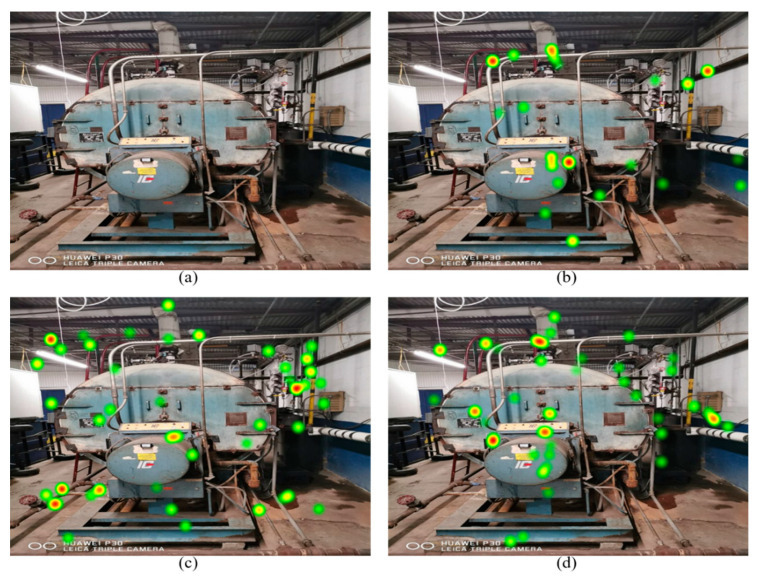
Attentional distribution (heat map): (**a**) original image; (**b**) participant before receiving the in-class training; (**c**) participant after receiving the in-class training; (**d**) experienced participant.

**Figure 5:**
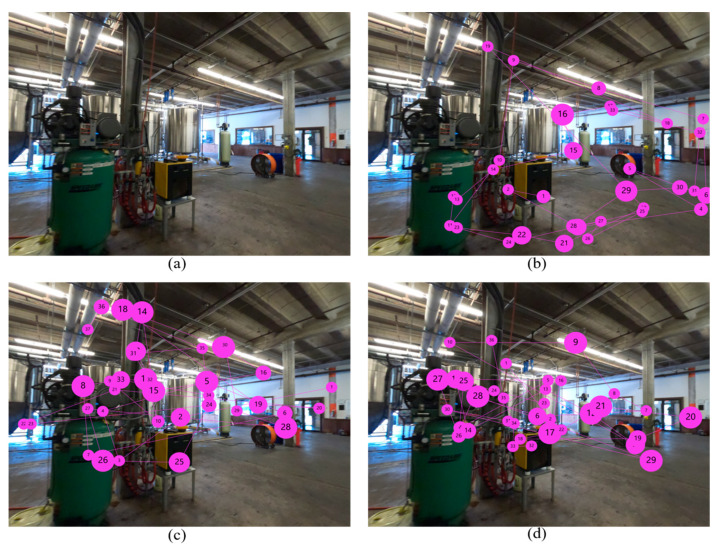
Search pattern (gaze plot): (**a**) original image; (**b**) participant before receiving the in-class training; (**c**) participant after receiving the in-class training; (**d**) experienced participant.

The depicted attentional distributions (Figure 4) show that subjects pay more attention to energy-saving opportunities after receiving the in-class training. Moreover, heat map comparisons indicated that participants who received the in-class training demonstrated similar attentive behavior toward areas of interest compared with experienced participants. Figure 5 shows the search patterns of participants using the gaze plot. A gaze plot is a useful tool for discovering the participants’ eye path and cognitive process. The scan paths also validate the results obtained through statistical analysis, which demonstrates more attention to energy-saving opportunities after receiving the in-class training by participants. Additionally, the search strategy of participants who received in-class training was similar to experienced participants.

## 4. Conclusions

Recognizing progress in employees’ performance after the training helps companies to identify the profit obtained from investing in human resources. One of the methods of measuring training performance is to evaluate attention behavior during work. There is a direct link between attention and eye-movement pattern; therefore, evaluating visual behavior is a valid method to study attention. This study presented an approach to evaluate the effectiveness of industrial energy assessment training using the eye-tracker technology. The training program was designed to enhance the knowledge of participants in recognizing the opportunities for potential energy saving in the manufacturing facilities.

The results showed that, after receiving the training, the subjects’ performance in energy assessment improved significantly in two areas: production and recycling and waste management. Moreover, the designed training program enhanced the knowledge of participants in recognizing energy-saving opportunities to the knowledge level of experienced participants. Using a neurophysiological device such as the eye-tracker in this study helps provide more accurate data related to knowledge enhancement than self-reported methods because eye-trackers capture the eye movement of participants in experiments and reflect their cognitive behavior.

The majority of eye-tracking studies were on static images, and a common task is observing pictures or video clips in a lab environment. Participants were asked to sit on a chair and look at targets on a screen. Then, their eye movement was tracked using an eye-tracker. These laboratory studies may not consider distracting features that are present in the real world, such as other people, devices, and eye-catching objects. They do not even assess the impact of safety issues on visual behavior. This proposed study was also limited to data collection in a lab environment. Therefore, future studies in the real facility environment could provide a more accurate assessment of the industrial energy assessment training. Hence, participants can walk through the facility and face distracting features that may capture their attention to remove the limitation of the lab setting.

## Figures and Tables

**Table 1:** Standard metrics used for evaluating visual behavior.

Gaze Location	[10,12,22,23,24,25]
Pupil Dilation	[11]
Fixation Duration	[4,13,15,18,19,20,21,22,26]
Fixation Frequency	[4,13,14,16,18,19,21,25]
Saccade Duration	[4,13,20]
Saccade Frequency	[14,15,16,19]
Total Recording Duration	[19,26]
First Fixation Duration	[15]

**Table 2:** Proposed hypotheses.

Hypothesis	Null Hypothesis
H_1_^(1)^: In-class training improves students’ performance in energy assessment (comparing students’ performance before and after training)	H_0_^(1)^: In-class training has no impact on students’ performance in energy assessment.
H_1_^(2)^: Students who received training in the class (group A2) show the same or better performance in energy assessment as experienced people (group A3) do.	H_0_^(2)^: Students who received the in-class training (group A2) have the same performance in energy assessment compared with students who are experienced (group A3).

**Table 3:** Participants’ performance before and after in-class training.

Energy-Saving Opportunity Areas of Interest (AOIs)	Group	Number of Samples	Fixation Duration (s)		
			Mean	Standard Deviation	Median
Production	A1 (No Training)	11	2.247	1.09	2.068
	A2 (In-class Training)	11	3.375	1.06	3.345
Building and HVAC	A1 (No Training)	11	1.812	0.82	1.822
	A2 (In-class Training)	11	2.189	1.29	1.925
Recycling and Waste Management	A1 (No Training)	11	2.204	0.87	2.02
	A2 (In-class Training)	11	2.903	1.03	3.2

**Table 4:** Performance of experienced students (group A3) in energy assessment vs. performance of students who received in-class training (group A2).

Energy-Saving Opportunity AOIs	Group	Number of Samples	Fixation Duration (s)		
			Mean	Standard Deviation	Median
Production	A2 (In-class Training)	11	3.375	1.06	3.345
	A3 (Experienced)	4	2.977	1.46	2.596
Building and HVAC	A2 (In-class Training)	11	2.189	1.29	1.925
	A3 (Experienced)	4	2.098	0.96	2.154
Recycling and Waste management	A2 (In-class Training)	11	2.903	1.03	3.203
	A3 (Experienced)	4	1.676	0.4	1.539

**Table 5:** Normality results for groups participated in the experiment.

	Group A1 (No Training)		Group A2 (In-Class Training)		Group A3 (Experienced)	
Energy-Saving Opportunity AOIs	Shapiro–Wilk’s W	*p*-Value	Shapiro–Wilk’s W	*p*-Value	Shapiro–Wilk’s W	*p*-Value
Production	0.913	0.266	0.937	0.481	0.904	0.451
Building and HVAC	0.978	0.953	0.886	0.124	0.933	0.971
Recycling and Waste Management	0.882	0.112	0.931	0.421	0.832	0.173

**Table 6:** Levene’s test results for homogeneity of variances across the groups.

Energy-Saving Opportunity AOIs	Group A1 vs. Group A3 (No Training vs. Experienced)		Group A2 vs. Group A3 (In-class Training vs. Experienced)	
	Levene’s f	*p*-Value	Levene’s f	*p*-Value
Production	0.266	0.615	0.324	0.579
Building and HVAC	0.086	0.774	1.096	0.314
Recycling and Waste Management	3.044	0.105	2.179	0.164

**Table 7:** Student’s *t*-test results for comparing the performance of participants in recognizing energy-saving opportunities.

Energy-Saving Opportunity AOIs	Group A1 vs. Group A2 (No Training vs. In-Class Training)		Group A2 vs. Group A3 (In-Class Training vs. Experienced)	
	*t*-Test	*p*-Value ^1^	*t*-Test	*p*-Value ^2^
Production	2.465	0.011	0.585	0.568
Building and HVAC	0.817	0.211	0.127	0.9
Recycling and Waste Management	1.721	0.05	2.275	0.04

^1^ One-tailed *p*-value, ^2^ two-tailed *p*-value.

## Data Availability

The data presented in this study are available on request from the corresponding author. The data are not publicly available due to data privacy.
